# Loss of TARBP2 Drives the Progression of Hepatocellular Carcinoma *via* miR-145-SERPINE1 Axis

**DOI:** 10.3389/fonc.2021.620912

**Published:** 2021-06-24

**Authors:** Li-Man Li, Chang Chen, Ruo-Xi Ran, Jing-Tao Huang, Hui-Lung Sun, Chang Zeng, Zhou Zhang, Wei Zhang, Song-Mei Liu

**Affiliations:** ^1^Department of Clinical Laboratory, Center for Gene Diagnosis, and Program of Clinical Laboratory Medicine, Zhongnan Hospital of Wuhan University, Wuhan, China; ^2^Department of Preventive Medicine, Northwestern University Feinberg School of Medicine, Chicago, IL, United States; ^3^Department of Clinical Laboratory, Renmin Hospital, Wuhan University, Wuhan, China; ^4^Department of Chemistry and Institute for Biophysical Dynamics, Howard Hughes Medical Institute, The University of Chicago, Chicago, IL, United States; ^5^Institute of Precision Medicine, Jining Medical University, Jining, China; ^6^Hubei Province Key Laboratory of Allergy and Immunology, Wuhan, China

**Keywords:** HCC, TARBP2, miR-145, SERPINE1, progression

## Abstract

The clinical outcomes of hepatocellular carcinoma (HCC) remain dismal. Elucidating the molecular mechanisms for the progression of aggressive HCC holds the promise for developing novel intervention strategies. The transactivation response element RNA-binding protein (TRBP/TARBP2), a key component of microRNA (miRNA) processing and maturation machinery has been shown to play conflicting roles in tumor development and progression. We sought to investigate the expression of *TARBP2* in HCC using well-characterized HCC cell lines, patient-derived tissues and blood samples. Additionally, the potential prognostic and diagnostic value of *TARBP2* in HCC were analyzed using Kaplan-Meier plots and ROC curve. Cell counting kit‐8 (CCK‐8), wound healing and transwell assays examined the ability of *TARBP2* to induce cell proliferation, migration, and invasion in HCC cell lines. RNA sequencing was applied to identify the downstream elements of *TARBP2*. The interaction of potential targets of *TARBP2*, miR‐145 and serpin family E member 1 (*SERPINE1*), was assessed using luciferase reporter assay. *TARBP2* expression was down-regulated in HCC cell lines relative to normal hepatocyte cells, with a similar pattern further confirmed in tissue and blood samples. Notably, the loss of *TARBP2* was demonstrated to promote proliferation, migration, and invasion in HCC cell lines. Interestingly, the reduction of *TARBP2* was shown to result in the upregulation of *SERPINE1*, also known as plasminogen activator inhibitor (*PAI-1*), which is a vital gene of the HIF-1 signaling pathway. Knockdown of *SERPINE1* rescued the *TARBP2*-lost phenotype. Moreover, *TARBP2* depletion induced the upregulation of *SERPINE1* through reducing the processing of miR-145, which directly targets *SERPINE1*. Finally, overexpression of miR-145 repressed *SERPINE1* and rescued the functions in sh-*TARBP2* HCC cells. Our findings underscore a linear TARBP2-miR-145-SERPINE1 pathway that drives HCC progression, with the potential as a novel intervention target for aggressive HCC.

## Introduction

Liver cancer is predicted to be the sixth most common cancer and the fourth leading cause of cancer deaths worldwide, with about 841,000 new cases and 782,000 deaths annually, according to the 2018 Global Cancer Statistics (1). Hepatocellular carcinoma (HCC) comprises 75%-85% of all liver cancer cases (1). HCC is often diagnosed at advanced stages and is highly resistant to conventional chemotherapy (2). Although advances in cancer care and treatment have improved patient survival, the clinical outcomes of HCC remain dismal, with a less than 50% five-year survival rate after diagnosis (3). The development of HCC is driven by various risk factors, including alcohol abuse, metabolic syndromes, hepatitis B virus (HBV) (4, 5), and hepatitis C virus infection. Epigenetic regulators, including microRNAs (miRNAs) (2), DNA methylation, hydroxymethylation and N6-methyladenosine (m^6^A), have also implicated in the development of HCC (5–9). Recent technological breakthroughs have also enabled exploiting epigenetic modifications as noninvasive biomarkers for early detection of HCC, showing potential to outperform or supplement α-fetoprotein (AFP) (7, 8, 10). Besides detection of HCC patients at early stage, enhancing our understanding of the molecular mechanism underlying the progression of aggressive HCC could lead to novel intervention strategies for improving the clinical outcomes of HCC.

The multidomain human TAR element binding protein (TRBP/TARBP2), containing three double-stranded RNA-binding domains, is a component of the Dicer-containing complex. Dicer-TRBP processes precursor miRNAs to generate mature miRNAs (11). TRBP also mediates miRNA isoform processing to generate related miRNAs of altered targeting specificity and differing lengths (12). As a Dicer partner protein, TRBP maintains miRNA production and subsequent gene silencing (13). Though TARBP2 has been involved in critical biological processes and pathogenesis, its expression patterns and functions as a tumor suppressor or promoter have been conflicting in different cancers, such as prostate cancer, gastrointestinal cancer, breast cancer, diffuse large B-cell lymphoma, and cutaneous malignant melanoma (14). In particular, the roles of *TARBP2* in HCC progression and its regulating pathway have not been established.

Aberrant miRNA expression could drive HCC development and global miRNA downregulation at a later stage could promote metastasis (2). Previous report has suggested that depletion of *TARBP2* resulted in reduction of a number of miRNAs including miR-145 (15). Overexpression of miR-145 was also found to inhibit the proliferation of HepG2 cells (16). As a target gene of miR-145, serpin family E member 1 (*SERPINE1*) (17) has shown increasing prominence in the development from chronic hepatitis to HCC. Besides, up-regulation of *SERPINE1* promoted HCC progression (18, 19). Thus, our hypothesis is that *TARBP2* modulates HCC development *via* regulation of miRNA biogenesis. We collected patient-derived blood and tissue samples to investigate whether *TARBP2* was dysregulated in HCC patients and correlated with prognosis. *TARBP2* loss/gain-of-function experiments in well-characterized HCC cell lines were then performed to confirm the observations found in clinical samples. We further utilized RNA-sequencing (RNA-Seq) to identify the potential downstream gene players and their functional relevance. Our findings provided novel insights into the link between *TARBP2* loss-of-function and HCC cells proliferation, migration, invasion and revealed a linear TARBP2-miR-145-SERPINE1 axis important to HCC progression.

## Materials and Methods

### Study Population and Clinical Parameters

We collected 52 pairs of HCC tumor and adjacent normal tissue samples, and 156 peripheral blood samples, including newly diagnosed HCC patients (N = 86) and healthy controls (N = 70) from Zhongnan Hospital, Wuhan University between May 2016 and February 2019. All blood samples were collected at the time of diagnosis, before surgery or other radio-/chemotherapy. HCC diagnosis was confirmed by pathologists according to the Guidelines for Diagnosis and Treatment of Primary Liver Cancer in China (2017 Edition). Tumor stages were defined according to the Barcelona Clinic Liver Cancer (BCLC) staging system (20). Healthy controls were recruited from those individuals who underwent regular physical examinations at Zhongnan Hospital, and individuals with severe chronic diseases (e.g. diabetes and hypertension) and/or a family history of cancer through questionnaires were excluded. Overall survival (OS) was defined as the interval between surgery and either death or the last observation taken. Serum biochemical parameters including alanine aminotransferase (ALT), aspartate aminotransferase (AST), glucose, blood urea nitrogen (BUN), creatinine (CREA), uric acid, total cholesterol (TC), triglyceride (TG), high-density lipoprotein cholesterol (HDL-C), low-density lipoprotein cholesterol (LDL-C) were measured using an automatic chemistry analyzer (AU5831, Beckman Coulter, Brea, CA, USA) with commercial kits. Serum tumor biomarkers including AFP and carcinoembryonic antigen (CEA) were measured using a chemiluminescence immunoanalyzer (i2000, Abbott Laboratories, Chicago, IL, USA) according to conventional protocols. Informed consent was obtained from each participant. This study was approved by the Ethics Committee of Zhongnan Hospital, Wuhan University (Approval Number: 2017058).

### Cell Culture

Human liver cell line HL7702 (L02), HCC cell lines (Hep3B, SNU387, MHCC97H and HepG2), and HEK-293T cells (**Table S1**) were purchased from Shanghai Institutes of Biological Sciences, Chinese Academy of Sciences (Shanghai, China). All cells were cultured in DMEM or MEM (HyClone, Fisher Scientific, Pittsburgh, PA, USA) with 10% fetal bovine serum (FBS) (Biological Industries, Beit Haemek, Israel) in 5% CO_2_ at 37°C according to instructions. The MHCC97H cells with high metastatic potential and the well-known HepG2 cells were used to illucidate tumor biological functions. Specifically, each experimental group and the corresponding control group were performed in the same batch.

### RNA Extraction and qRT-PCR

Total RNA was extracted from tissues, white blood cells or cultured cells using the TRIzol reagent (Invitrogen, Life Technologies, Carlsbad, CA, USA). The cDNA was synthesized using the Reverse-Transcription Ace qPCR RT Master Mix with gDNA Remover Kit (Takara, Kyoto, Japan). The mRNA expression was evaluated in triplicates using qRT-PCR. The primers are listed in **Table S2**.

### Selection of Potential miRNAs

The miRTarBase (21) and TargetScanHuman (22) were used to predict potential miRNA targets. We selected final targets according to the prediction results from these public databases and previous reports (23–27).

### Measurement of miRNA Transcription

Reverse transcriptions were performed using the All-in-One™ miRNA First-Strand cDNA Synthesis Kit (GeneCopoeia, Rockville, MD, USA). The All-in-One™ miRNA qRT-PCR Detection Kit (GeneCopoeia, Rockville, MD, USA) was used to quantify miR-30b-5p (HmiRQP0393), miR-30c-5p (HmiRQP0396), miR-192-5p (HmiRQP0274) and miR-145-5p (HmiRQP0192) in tissues and cell lines. The hsnoRNA-U48 (HmiRQP9021) was served as the control.

### Transfection Assay

Lentiviruses containing *TARBP2* overexpression (oe)/short hairpin (sh) RNAs/controls (Ctrl) were produced in HEK-293T cells with packaging vectors pCMV delta R8.2 and pCMV-VSV-G (from Dr. Chuan He’s Laboratory, Department of Chemistry, The University of Chicago, IL, USA). The medium supernatants were collected and concentrated by PEG-it Virus Precipitation Solution (SBI, System Biosciences, Palo Alto, CA, USA). The obtained viruses were then used to infect MHCC97H and HepG2 cells after 48 hr, oe/sh-*TARBP2* or oe/sh-Ctrl stable cell lines were selected by 2 µg/mL puromycin. Transient transfections of plasmids (*SERPINE1* shRNA and scramble control) (Genechem, Shanghai, China) were performed with Lipofectamine 3000 (Invitrogen, Life Technologies, Carlsbad, CA, USA). Approximately 2.5×10^6^ cells were transfected with 2.5 μg of plasmid using 3.75 μL of Lipofectamine 3000. The miR-145 mimic and its negative control (NC) used for transfection were purchased from Ribobio (Guangzhou, China). Transfections were performed using the riboFECT™ CP Kit (Ribobio, Guangzhou, China).

### Western Blot Analysis

Cultured cells were lysed by RIPA buffer (Beyotime, Shanghai, China) containing protease inhibitor (Roche, Eugene, OR, USA). Protein concentrations were determined by BCA Protein Assay Kit (Beyotime, Shanghai, China). Extracted proteins were separated by 10% SDS-PAGE and transferred to polyvinylidene fluoride (PVDF) membranes (Millipore, Billerica, MA, USA). The membranes were blocked in 5% non-fat milk followed by overnight incubation with primary antibodies including rabbit anti-TRBP (15753-1-AP; ProteinTech, Wuhan, China), rabbit anti-PAI-1 (SERPINE1) (13801-1-AP; ProteinTech, Wuhan, China) and rabbit anti-alpha TUBULIN (11224-1-AP; ProteinTech, Wuhan, China). After washing for three times, the membranes were incubated with the secondary antibody. The signals were detected by the Odyssey^®^ CLx IRImaging System (LI-COR Biosciences, Lincoln, NE, USA).

### Cell Proliferation Assay

Transfected cells were seeded in 96-well plates (MHCC97H: 4,000 cells/well; HepG2: 3,000 cells/well). After incubation for 0, 1, 2, 3, 4 and 5 days, respectively, 10 μL of Cell Counting Kit-8 (Dojindo, Kumamoto, Japan) reagent was added to each well and incubated for 1.5 hr at 37°C. The absorbance was measured at 450 nm with a microplate reader (PerkinElmer, Fremont, CA, USA).

### Migration and Invasion Assays

Invasion assay was carried out using 24-well plates with the Matrigel coating (BD, Bioscience, Franklin Lakes, NJ, USA). The migration assay was carried out without the Matrigel coating. Transfected cells (MHCC97H: 2×10^4^ cells/chamber; HepG2: 8×10^4^ cells/chamber) were seeded in the upper chamber of a transwell (BD, Bioscience, Franklin Lakes, NJ, USA) containing serum-free medium. DMEM or MEM medium containing 10% FBS was placed in the lower chamber. After incubation for 24 hr (MHCC97H) or 48 hr (HepG2), migrated or invaded cells were fixed with 4% paraformaldehyde for 15 min and stained with 0.1% crystal violet for 20 min. Cells were imaged with a fluorescence microscope (Olympus, CKX41, Tokyo, Japan).

### Colony Formation Assay

Transfected cells (1,000 cells/well) were seeded into 6-well plates (Corning Costar, Corning, NY, USA) and cultured in DMEM or MEM medium containing 10% FBS for approximately 2 weeks. The colonies were fixed with 4% paraformaldehyde for 15 min and stained with 0.1% crystal violet for 20 min.

### Wound Healing Assay

Transfected cells were seeded in 6-well plates and serum-starved for 48 hr. The wound was scratched by a 200 μL-yellow-tip and the pictures were taken at 0 hr and 48 hr. The closed areas were measured by the Image J software (National Institutes of Health, Bethesda, MD, USA) and the wound healing rate (WHR) was calculated as follow: WHR (%) = (Closed area)/(Initial area) ×100%.

### Cell Cycle Assay

Transfected cells were harvested and washed with pre-cold PBS. Then 10 μL permeabilization solution and 1 mL 1× DNA staining solution containing propidium iodide (Multi Sciences, Hangzhou, China) were used to resuspend cell pellets. Cell resuspensions were incubated in the dark at room temperature for 30 min. The cell cycle distributions of stained cells were assessed by flow cytometry (Beckman Coulter, Brea, CA, USA).

### RNA Sequencing and Data Processing

The concentration and purity of RNA for oe/sh-*TARBP2* HepG2 cells were detected before the RNA-Seq. The paired-end (150 bp) RNA-Seq was performed by GENEWIZ (Suzhou, China) using the Illumina HiSeq 4000 Sequencer (Illumina, Inc., USA) according to Illumina’s instructions. Raw sequencing reads were trimmed by FASTX-Toolkit (v0.0.14) to remove the adaptor sequences. The reads were aligned to Ensembl human genome reference hg19 using STAR (v2.7.3) (28) with end-to-end alignment mode. Alignments and counting of gene bodies were conducted by featureCounts from R package Rsubread (v1.30.9) without strand information. The quantile normalization among all genes, and the fold change calculation were performed by Bioconductor tools within the R Statistical Package (v3.6.1) (29). Differential genes between cell lines were evaluated based on fold change at 70% cut-off. The NIH/DAVID tool was used to explore functional relevance of differential genes for Gene Ontology biological processes and canonical pathways (30, 31). The raw and processed RNA-Seq data have been uploaded to the NCBI Gene Expression Omnibus database (Accession Number: GSE143551).

### Luciferase Reporter Assay

The 3’-UTR (untranslated region) fragment of the wild-type *SERPINE1* containing the putative miR-145 binding site and the mutant *SERPINE1* sequence were cloned into pmirGLO luciferase plasmid respectively (Promega, Madison, WI, USA). HepG2 cells were transfected with different reporter vectors (pmirGLO-*SERPINE1*-wild-type and pmirGLO-*SERPINE1*-mutant) and co-transfected with the negative control (NC) or miR-145 mimic. Luciferase reporter assay was performed 48 hr after transfection using the Dual-Luciferase Kit (Promega, Madison, WI, USA).

### Statistical Analysis

All data was computed using the SPSS package (v20.0, SPSS, Chicago, IL, USA). The Student’s t-test or Mann-Whitney test, paired t-test, or Fisher’s exact test were applied to compare the difference between groups. The Receiver Operating Characteristic (ROC) curve was used for the evaluation of diagnostic performance. Pearson correlation or Spearman correlation was used for the correlation analysis. The Kaplan-Meier (KM) test was performed to estimate the OS of HCC patients. The p-value < 0.05 was considered as statistically significant.

## Results

### Downregulation of *TARBP2* in HCC Is Associated With Poor Prognosis

**Figure 1:**
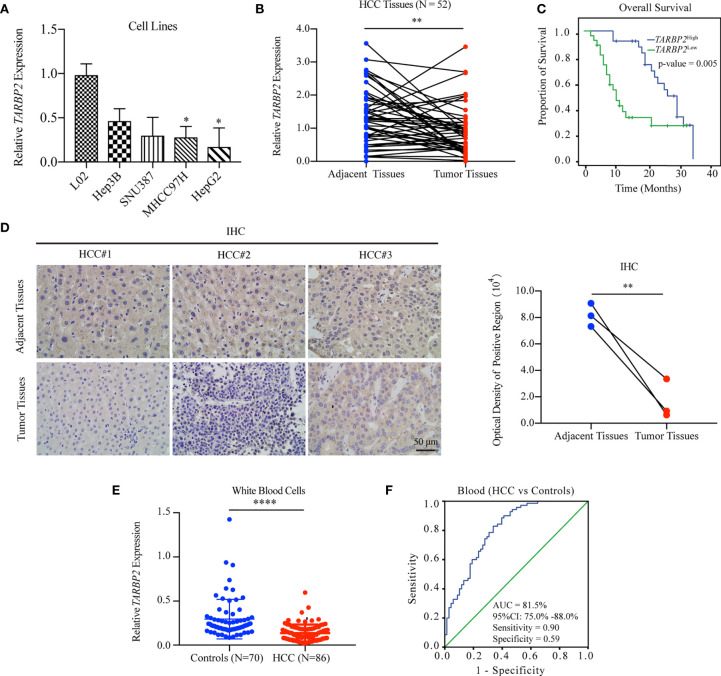
Downregulation of TARBP2 in HCC is associated with poor prognosis. **(A)** The mRNA expression of *TARBP2* was consistently downregulated across HCC cell lines compared with the normal liver L02 cells. **(B)** Downregulation of *TARBP2* mRNA was confirmed in 52 pairs of patient-derived HCC tumors and adjacent tissues. **(C)** HCC patients with a relatively lower mRNA expression of *TARBP2* had a worse three-year OS. **(D)** The protein expression of TARBP2 was higher in adjacent tissues relative to tumors. **(E)** The mRNA expression of *TARBP2* in patient-derived blood samples was lower relative to healthy controls. **(F)** *TARBP2* mRNA expression in the blood distinguished HCC patients from healthy controls. OS, overall survival; IHC, immunohistochemistry; AUC, area under curve; CI, confidence interval; *p-value < 0.05; **p-value < 0.01; ****p-value < 0.0001.

Comparison of the expression of *TARBP2* in a panel of liver cell lines showed that *TARBP2* was consistently downregulated across HCC cell lines compared with the normal liver L02 cells (**Figure 1A**). Downregulation of *TARBP2* was confirmed in 52 pairs of patient-derived HCC tumors and adjacent tissues (**Figure 1B**; paired t-test p-value = 0.0045). For the tissue samples from patients with clinical outcomes, the KM analysis indicated that HCC patients with a relatively lower expression of *TARBP2* had a worse three-year OS (**Figure 1C**; log rank p-value = 0.005). The immunohistochemistry (IHC) assay showed that TARBP2 protein was higher in adjacent tissues relative to tumors (**Figure 1D**; paired t-test p-value = 0.004). Besides, the protein level of TARBP2 was also analyzed based on Human Protein Atlas database (**Figure S1**). In addition, the expression of *TARBP2* in patient-derived white blood cells (N = 86) was significantly lower relative to healthy controls (N = 70) (**Figure 1E**); Mann-Whitney test p-value = 1.46×10^-11^. Collectively, these data demonstrated that *TARBP2* and its protein was decreased in tumor tissues and blood from HCC patients or in HCC cell lines, consistent with previous observations (32).

Specifically, *TARBP2* in white blood cells showed a good capacity for distinguishing HCC patients from healthy controls (area under curve [AUC] = 81.5%; 95% confidence interval [CI], 75.0%-88.0%) with a sensitivity of 0.90 and a specificity of 0.59 at the cut-off of 0.14 (**Figure 1F**). Additionally, *TARBP2* expression in tumor tissues showed a trend of association with the BCLC stage (Chi-square test p-value = 0.029) and portal vein tumor thrombus (PVTT) (Chi-square test p-value = 0.036) (**Table S3**). Next, we found that TARBP2 expression was lower in patients with BCLC stages B/C relative to patients with BCLC stage A (**Figure S2A**). In contrast, there was no difference between patients with and without PVTT (**Figure S2B**).

### Expression of *TARBP2* Reflects Clinical Parameters in Patient-Derived Serum

We collected the peripheral blood samples from 86 HCC patients and 70 healthy controls and compared their differences of clinical characteristics in serum. In HCC patients, ALT, AST, glucose, TC, AFP and CEA levels in serum were significantly higher than healthy controls (p-values < 0.05). In contrast, HDL-C was lower compared with healthy controls (p-value = 3.00×10^-7^) (**Table S4**). In addition, the expression of *TARBP2* was negatively associated with glucose (Pearson r = -0.30, p-value = 0.010) and positively associated with HDL-C (Pearson r = 0.27, p-value = 0.040) (**Figures S3A–D**).

### *TARBP2* Affects Proliferation, Migration, Invasion, and Cell Cycle in HCC Cell Lines

**Figure 2:**
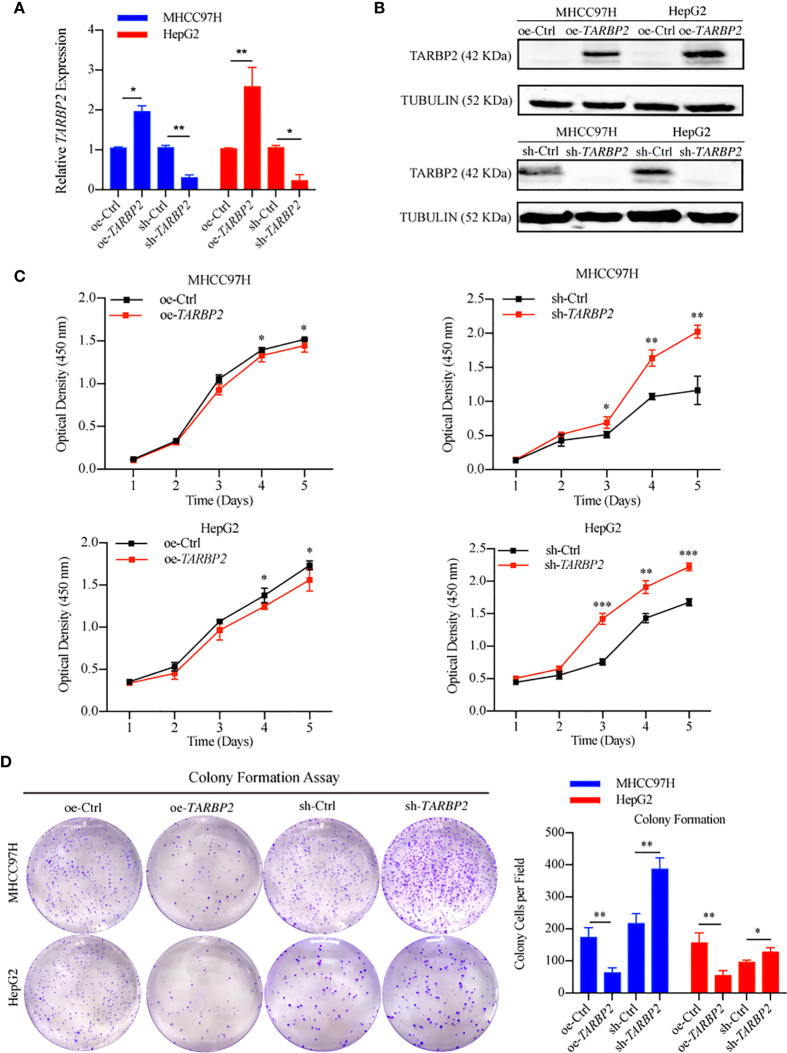
*TARBP2* affects proliferation of HCC cells. **(A, B)** The changes of mRNA and protein expression of *TARBP2* compared with controls in HCC cell lines after transfection. **(C, D)** oe-*TARBP2* HCC cells could inhibit proliferation, whereas sh-*TARBP2* HCC cells could promote proliferation. *p-value < 0.05; **p-value < 0.01; ***p-value <0.001.

**Figure 3:**
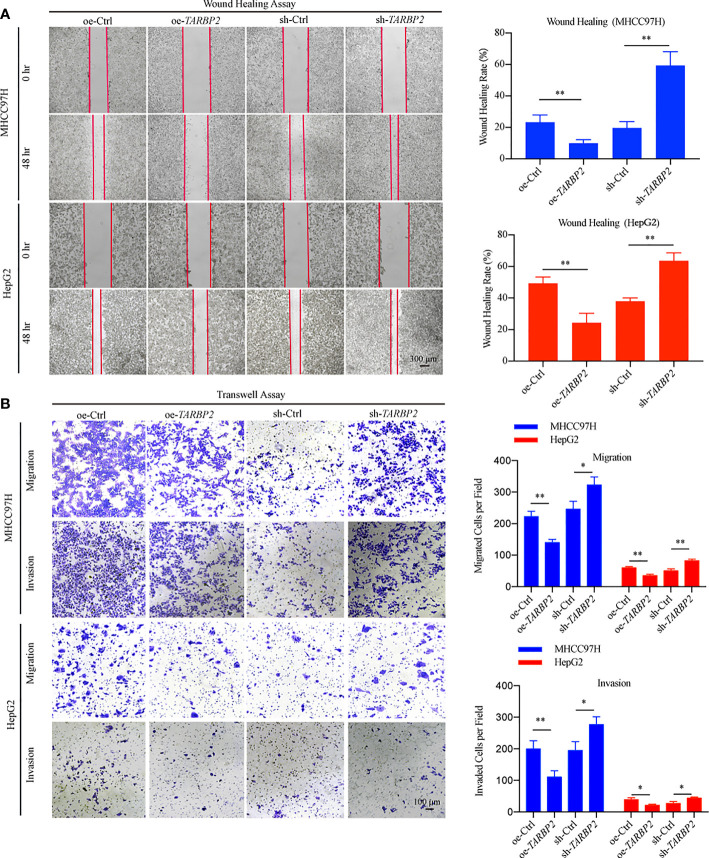
*TARBP2* affects migration and invasion of HCC cells. **(A, B)** *TARBP*2 overexpression suppressed the migration and invasion of the HCC cells compared with controls. *TARBP2* knockdown shown an opposite effect according to the results of wound healing and transwell assays. *p-value < 0.05; **p-value < 0.01.

To explore *TARBP2* loss/gain-of-function in HCC, we established stable oe/sh-*TARBP2* MHCC97H and HepG2 cell lines. The qRT-PCR and western blot verified the significant changes of mRNA and protein expression of *TARBP2* compared with oe/sh-Ctrl after transfection (**Figures 2A, B**). Based on colony formation assay and CCK-8 assay, the results indicated that overexpression of *TARBP2* could inhibit HCC cell proliferation, whereas *TARBP2* knockdown could promote proliferation (**Figures 2C, D**). *TARBP2* overexpression was also found to suppress the migration and invasion of the HCC cells compared with oe-Ctrl group. In contrast, *TARBP2* knockdown showed an opposite effect according to the results of wound healing and transwell assays (**Figures 3A, B**). The results of flow cytometry demonstrated that *TARBP2* overexpression increased the proportion of G1 phase, while *TARBP2* knockdown had a reverse effect on cell cycle (**Figure S4**).

### SERPINE1 Is a Downstream Player of *TARBP2*

**Figure 4:**
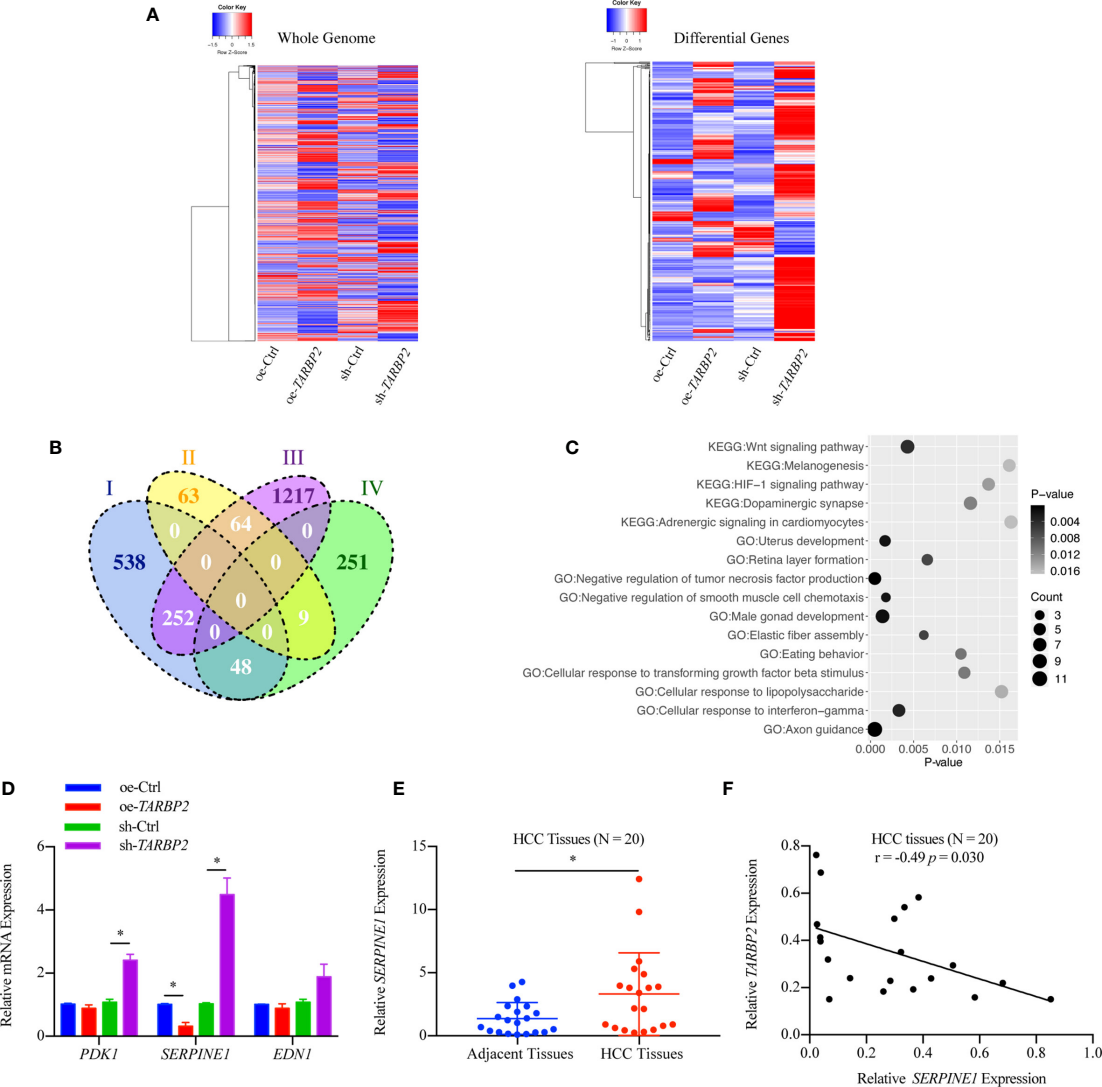
Differential genes after overexpression and knockdown of *TARBP2* in HepG2 cells. **(A)** Heatmaps represent the expression level of the whole genome (left) and showing the intersect of differentially expressed genes at 70% fold-change from two groups (right) respectively. (oe-Ctrl vs oe-*TARBP2*, and sh-Ctrl vs sh-*TARBP2*). **(B)** A Venn Diagram of differentially expressed genes at the cut-off of 70% fold-change. Group-I, up-regulated genes from oe-Ctrl to oe-*TARBP2*; Group-II, down-regulated genes from oe-Ctrl to oe-*TARBP2*; Group-III, up-regulated genes from sh-Ctrl to sh-*TARBP2*; Group-IV, down-regulated genes from sh-Ctrl to sh-*TARBP2*. **(C)** The KEGG pathways and GO biological processes enriched in the differentially expressed genes at 70% fold-change. **(D)** The mRNA expression levels of candidate genes from enriched pathway. **(E)** Relative expression levels of *SERPINE1* in HCC tissues. **(F)** Correlation between the relative expression level of *TARBP2* and *SERPINE1* in HCC tissues. *p-value < 0.05.

RNA-Seq in the oe-*TARBP2* HepG2 cells identified 136 genes down-regulated and 838 genes up-regulated after overexpression of *TARBP2* (fold change > 70%). In contrast, 308 genes were down-regulated and 1533 genes were up-regulated after knockdown of *TARBP2* in HepG2 cells (fold change > 70%). Transcriptomic expression levels of the whole genome and the differentially expressed genes at the cut-off of 70% fold change were displayed (**Figure 4A**). At the fold change of 70%, the Venn diagram showed 373 shared genes between the differentially expressed genes in oe- and sh-*TRABP2* HepG2 cells (**Figure 4B**). The KEGG and GO analysis revealed enriched pathways and biological processes relevant to cancer pathobiology, including such pathways as “Wnt signaling pathway”, “HIF-1 signaling pathway”, and “Adrenergic signaling in cardiomyocytes”; as well as such biological processes as “Negative regulation of tumor necrosis factor production”, “Axon guidance”, and “Cellular response to interferon-gamma” (**Figure 4C**). Notably, the HIF-1 signaling pathway was consistently enriched under different cut-offs of differential expressed genes, from 50% to 80% fold changes (**Table S5** and **Table S6**). Among the differential genes, phosphoinositide-dependent protein kinase-1 (*PDK1*), endothelin 1 (*EDN1*) and *SERPINIE1* are vital regulators of the HIF-1 signaling pathway (**Table S5**). The viability and migration of hepatic cells could be suppressed by the inhibition of PDK1 activity (33). Lu and colleagues have conducted liver up-regulated EDN1 expression in zebrafish, which leads to steatosis, fibrosis, and HCC (34). *SERPINE1* contributed to the development of HCC (35) acting as a mediator both in two typical pathways, HIF-1 pathway and P53 pathway in HCC (**Figures S5**-**S6**). Mechanically, the heterodimeric transcription factor HIF-1 was activated under hypoxic conditions, leading to the upregulation of its target gene PAI-1 (SERPINE1) (36).

Therefore, we verified the mRNA expression of *PDK1*, *EDN1* and *SERPINE1* using qRT-PCR. As a result, *SERPINE1* was down-regulated in the oe-*TARBP2* HepG2 cells and was up-regulated in the sh-*TARBP2* HepG2 cells (**Figure 4D**). Importantly, *SERPINE1* was found to be increased in HCC tumors relative to their adjacent tissues (N = 20) (paired t-test p-value = 0.038) (**Figure 4E**), consistent with a previous study (18). The expression of *SERPINE1* was also shown to be inversely correlated with *TARBP2* expression in HCC tissues (Pearson r = -0.49, p-value = 0.030) (N = 20) (**Figure 4F**).

**Figure 5:**
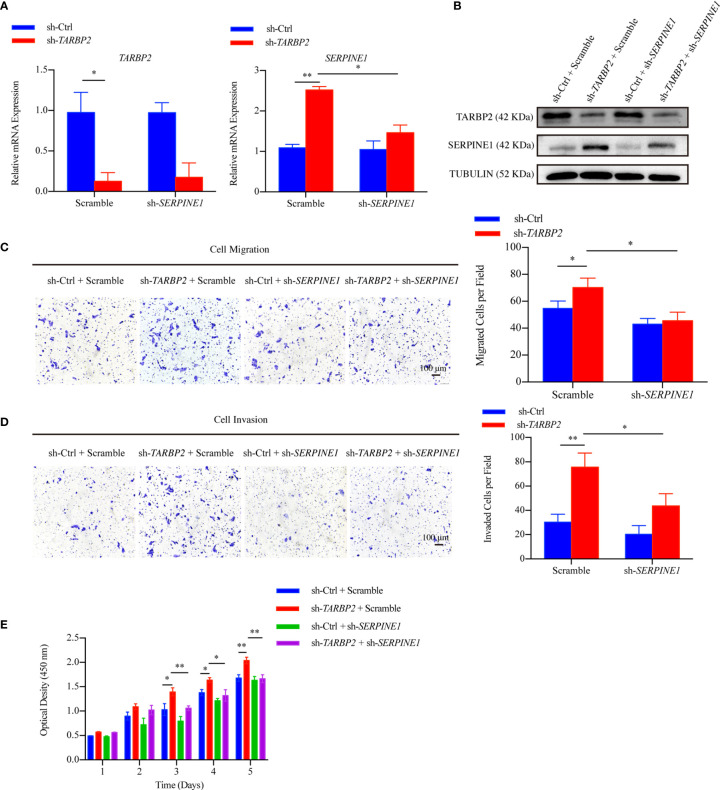
SERPINE1 is a downstream player of *TARBP2*. **(A, B)** The mRNA and protein expression of *TARBP2* and *SERPINE1* in the stable sh-*TARBP2* HepG2 cells after treatment with scramble control and sh-*SERPINE1*. **(C)** Cell migration; **(D)** Invasion; and **(E)** Proliferation assays of sh-*TARBP2* HepG2 cells treated with scramble control and sh-*SERPINE*1. *p-value < 0.05; **p-value < 0.01.

Then, we used specific shRNA to knockdown *SERPINE1* in the stable sh-*TARBP2* HepG2 cells (**Figures 5A, B**), predicting that it would suppress the promotion phenotype of sh-*TARBP2*. We noticed that the sh-*TARBP2* HepG2 cells treated with *SERPINE1* shRNA could rescue HepG2 cell migration (**Figure 5C**), invasion (**Figure 5D**), and proliferation (**Figure 5E**), whereas no significant changes were found in the scramble controls. The results from knockdown experiments were consistent with *in silico* prediction, illustrated that *SERPINE1* might be a downstream player of TARBP2.

### miR-145 Mediates *SERPINE1* to Affect the Role of *TARBP2* in HCC Progression

**Figure 6:**
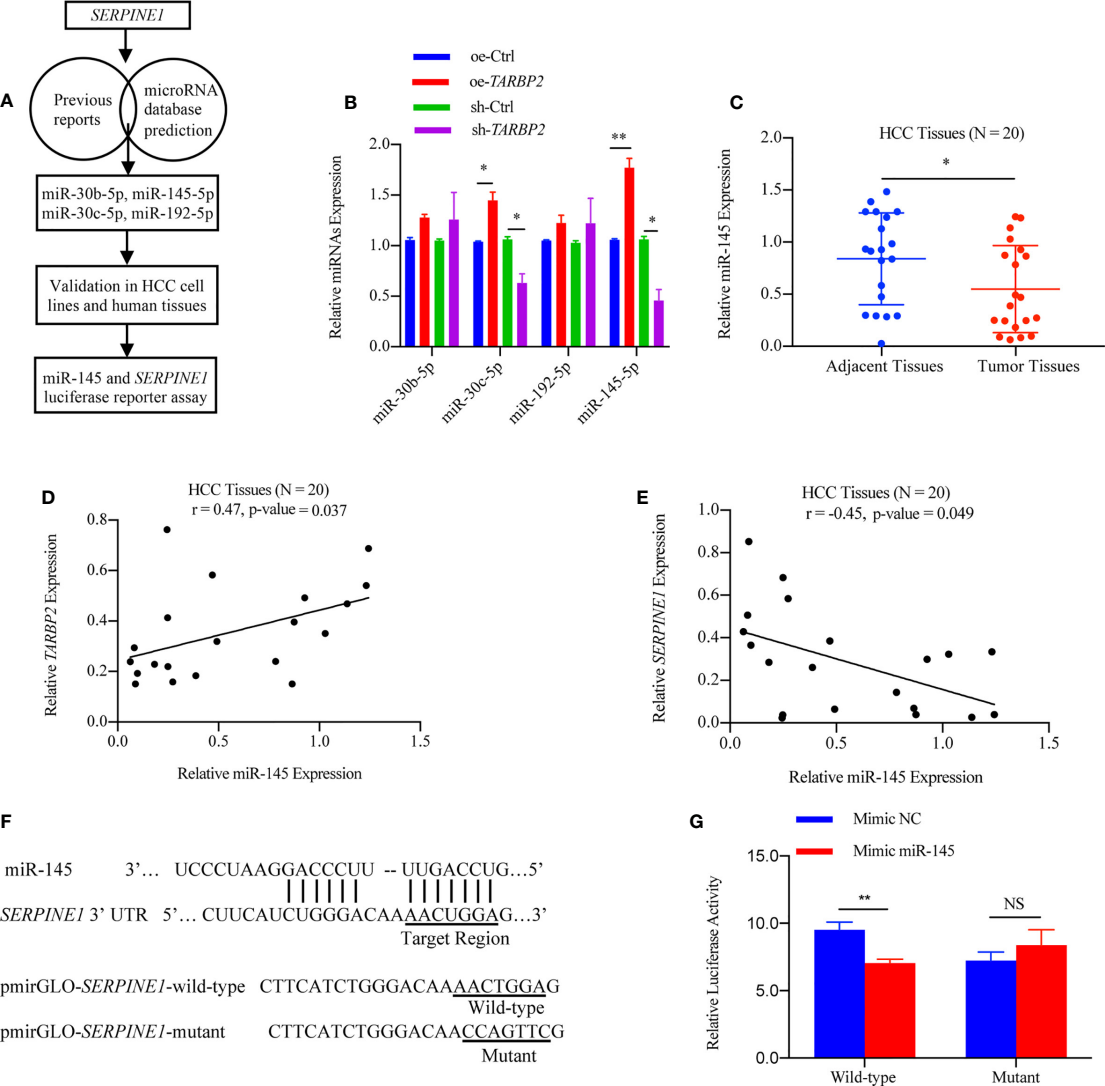
*SERPINE1* mRNA is a target of miRNA-145 linked to *TARBP2*. **(A)** Flow chart of selection and analysis of potential miRNAs. **(B)** Levels of 4 potential miRNAs in the oe/sh-*TARBP2* HepG2 cells. **(C)** Comparison of miR-145 expression between HCC tumors and tumor adjacent tissues (N = 20) (paired t-test p-value = 0.038). **(D, E)** miR-145 levels in HCC tumors are positively associated with *TARBP2* (Pearson r = 0.47, p-value = 0.037) and negatively associated with *SERPINE1* (Pearson r = -0.45, p-value = 0.049) (N=20). **(F)** The binding sites of miR-145 in the 3’-UTR of *SERPINE1* mRNA. **(G)** The effects of miR-145 on reporters, pmirGLO-*SERPINE1*-wild-type and pmirGLO-*SERPINE1*-mutant, were measured by luciferase reporter assays in HepG2 cells. *p-value < 0.05; **p-value < 0.01; NS, no significance; NC, negative control.

Considering that the decrease of TARBP2 expression could lead to a broad defect in miRNA maturation (37), the levels of potential miRNAs which might target *SERPINE1* based on previous reports and miRNA prediction database were also evaluated in the oe/sh-*TARBP2* HepG2 cells, including miR-30b, miR-30c, miR-192 and miR-145 (**Figure 6A**). These 4 miRNAs have been confirmed to target *SERPINE1* or to be affected by the dysregulation of TARBP2 in previous studies (15, 38). A panel of these mature miRNAs, miR-30b, miR-30c, miR-192 and miR-145, were assessed by qRT-PCR (**Figure 6B**). Notably, the levels of miR-30c and miR-145 were regulated by *TARBP2* (**Figure 6B**). Importantly, miR-145 has shown a negative regulation of *SERPINE1* in human endometriotic cell line (17), and the expression of mature miR-145 was decreased in *TARBP2*-depleted Ewing sarcoma family tumor cell lines (15). These evidences suggested miR-145 might also be a TARBP2-dependent miRNA and target SERPINE1 in HCC. In the following experiments, we examined the expression of miR-145 in clinical samples, showing consistent results as what was observed in HCC cells. The expression of miR-145 was down-regulated in HCC tumors relative to adjacent tissues (N = 20) (paired t-test p-value = 0.005) (**Figure 6C**). The miR-145 levels were positively correlated with *TARBP2* (Pearson r = 0.47, p-value = 0.037) and negatively correlated with *SERPINE1* (Pearson r = -0.45, p-value = 0.049) (N = 20) (**Figures 6D, E**). Significant changes were observed in the luciferase assay with pmirGLO-*SERPINE1*-wild-type HepG2 cells but not in the mutant cells, indicating a direct role of miR-145 in regulating *SERPINE1* expression (**Figures 6F, G**).

**Figure 7:**
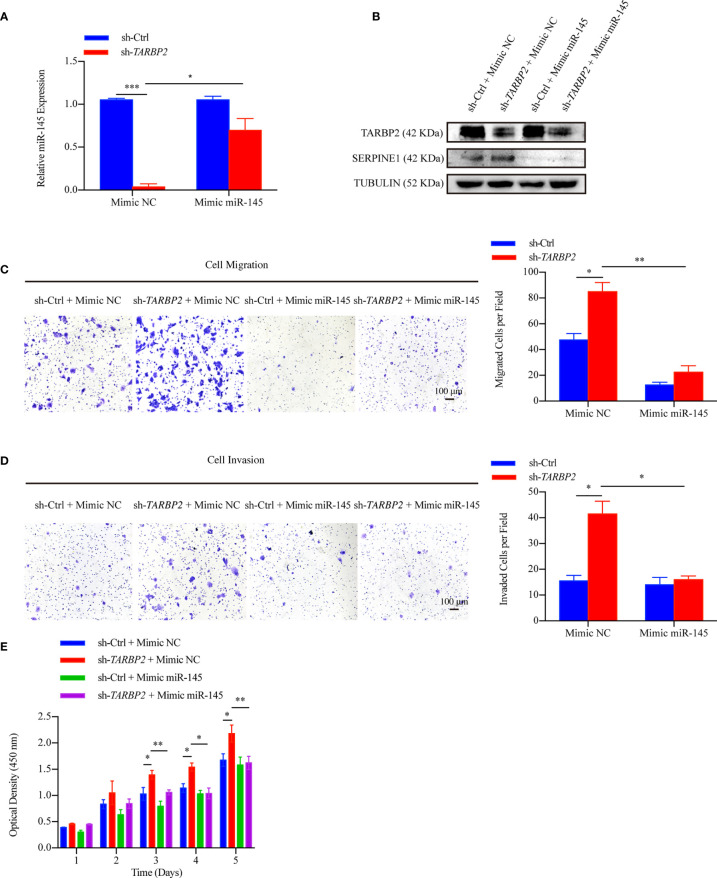
miR-145 mediates *SERPINE1* to affect the role of TARBP2 in HCC progression. **(A)** Levels of miR-145 in sh-*TARBP2* HepG2 cells after treatment with Mimic NC and Mimic miR-145. **(B)** Protein levels of SERPINE1 and TARBP2 in the stable sh-*TARBP2* HepG2 cells after treatment with Mimic NC and Mimic miR-145. **(C)** Cell migration; **(D)** Invasion; and **(E)** Proliferation of sh-*TARBP2* HepG2 cells treated with Mimic NC and Mimic miR-145. *p-value < 0.05; **p-value < 0.01; NC, negative control.

To further verify our hypothesis that up-regulation of *SERPINE1* was due to miR-145 downregulation, we restored miR-145 expression by mimic miR-145 in the stable sh-*TARBP2* HepG2 cells (**Figure 7A**). Consistent with our results of luciferase assay, *SERPINE1* protein was decreased compared with the NC groups (**Figure 7B**). Moreover, restoring miR-145 expression successfully rescued the migration (**Figure 7C**), invasion (**Figure 7D**), and proliferation (**Figure 7E**) in the stable sh-*TARBP2* HepG2 cells, while the NC control groups had no changes.

## Discussion

Although previous studies have implicated TARBP2 in various cellular processes such as miRNA maturation, or as a mediator for different pathways, its role in cancer development has been controversial and not been established in HCC yet. The lack of efficient intervention approaches has led to the unsatisfactory clinical outcomes of HCC, which represents a leading cause of cancer mortality worldwide. In the current report, we aimed to examine TARBP2 for its role in HCC progression and to explore related regulatory pathways, taking advantage of clinical specimens and well-characterized liver cancer/normal liver cell lines.

First, we sought to establish whether *TARBP2* was associated with HCC pathogenesis and patient survival. *TARBP2* and its protein were consistently found to be dysregulated in clinical specimens, including tumor/adjacent tissue samples and the peripheral blood derived from HCC patients and healthy controls. The molecular levels of *TARBP2* were explored to demonstrate its impact on the clinical outcomes in HCC patients, showing association with tumor stages and the OS of HCC patients within three-year after initial diagnosis, after controlling gender and age. Approximately 73.7% (14/19) of HCC patients with BCLC stage B and C as well as 76.5% (13/17) HCC patients with PVTT had a lower *TARBP2* expression (**Table S2**), demonstrating that *TARBP2* deficiency might drive HCC progression.

Aerobic glycolysis and mitochondrial dysfunction are key metabolic features of cancer cells (39). Liver is the main site of glucose and lipid metabolisms. Disturbed cholesterol and glucose promoted not only HCC but also cardiovascular diseases and other cancers (40). Hyperglycemia has been proven to accelerate tumorigenesis in HCC cells (41). Low levels of HDL-C have shown an association with the development of HCC (42). Consistent with previous reports (39–42), our data revealed that serum glucose was higher in HCC patients, while HDL-C was lower in HCC patients, compared to health controls. Moreover, the expression of *TARBP2* was positively associated with serum HDL-C and negatively associated with serum glucose (**Figures S3A–D**), indicating *TARBP2* might be involved in the disorders of glucose and lipids caused by liver dysfunction in HCC patients.

We further utilized a set of well-characterized HCC cell lines to demonstrate that TARBP2 affected cell proliferation, migration, invasion, and cell cycle. Our findings in clinical specimens and HCC cell lines established a critical role of TARBP2 in the progression of HCC, suggested a potential linkage between the molecular levels of TARBP2 and the clinical outcomes in HCC.

Second, in HCC cell lines, we aimed to illustrate the mechanism underlying the TARBP2-associated HCC progression. Examining RNA-Seq profiles in the oe/sh-*TARBP2* cell lines, several cancer-related pathways, such as the HIF-1 signaling pathway which played a pivotal role in HCC progression (43), were found to be significantly enriched among the differential genes in the cell lines, suggesting a systematic change in transcriptional profiles of various cancer-related genes after either overexpression or knockdown of *TARBP2*. Several functional genes involving the HIF-1 pathway were further evaluated in HCC cell lines and clinical specimens. As a result, the expression of *SERPINE1*, a vital element in both HIF-1 pathway and P53 pathway, was found to be increased in HCC tumors relative to their adjacent tissues, consistent with previous genomic and clinical studies in HCC (44, 45). Expression of *SERPINE1* was also shown to be inversely correlated with *TARBP2* expression in HCC tissues. All the above evidences suggested that *SERPINE1* might be a key downstream player of *TARBP2* and could mediate HCC progression. In addition, tumor biological functional experiments in sh-*TARBP2* HepG2 cells after restoring the expression of SERPINE1 further linked the observed up-regulation of *SERPINE1* in HCC with *TARBP2* as the potential downstream regulator.

Third, considering that *TARBP2* involves in multiple cellular processes, particularly its function in regulating the accuracy of miRNA processing (46), we aimed to elucidate an epigenetic pathway *via* miRNAs for the TARBP2-SERPINE1 axis in HCC progression. Our findings suggested that sh-*TARBP2* reduced the synthesis of mature miR-145, which have been identified to directly target *SERPINE1* in previous reports (17, 47, 48). Next, we validated the relationship between SERPINE1 and miR-145 in HCC using dual luciferase assays and clinical samples. Collectively, a working model was proposed to link TARBP2 and SERPINE1 through miR-145 in which miR-145 might serve as a potential downstream player of TARBP2 loss-of-function and directly modulate the expression and function of SERPINE1 in HCC progression.

We acknowledge that several limitations are noted for the current report. Since all HCC patients were from a Chinese cohort, the majority of which had a history of HBV infection. The relationship between TARBP2 and HCC progression in HCC of other risk background and populations will worth further investigation. Though the HCC cell lines used in this study have been well-characterized and widely used in previous studies, it is possible that these cell lines might contain unknown artifacts in cell culture that affected their transcriptional and/or epigenetic profiles. We showed that TARBP2 has an effect on progression of HCC, but whether the other classical proliferation, migration, and invasion markers, such as Ki-67, E-cadherin, vimentin, MMP7, MMP9, and Zeb1/2 could be affected in sh-TARBP2 and oe-TARBP2 cells was not assessed. Although we identified the TARBP2-dependent miR-145 based on sequencing data, online database and qRT-PCR, more comprehensive experiments such as Northern blots are warranted to validate our intriguing results in future. Our observations suggested the inhibition of TARBP2 function might constitute a critical component of the mechanism that underlies the defective mature miR-145 in HCC. However, the potential effect of TARBP2 on pre-miR-145 and the association between pre-miR-145 and miR-145 still remain to be clarified. Future studies using primary cell lines, and/or animal models as well as SERPINE1 rescue experiment will be helpful to confirm our findings in this report. Other key proteins such as DICER that are also involved in the miRNA machinery are potential areas in HCC that requires further research and validation.

## Conclusions

**Figure 8:**
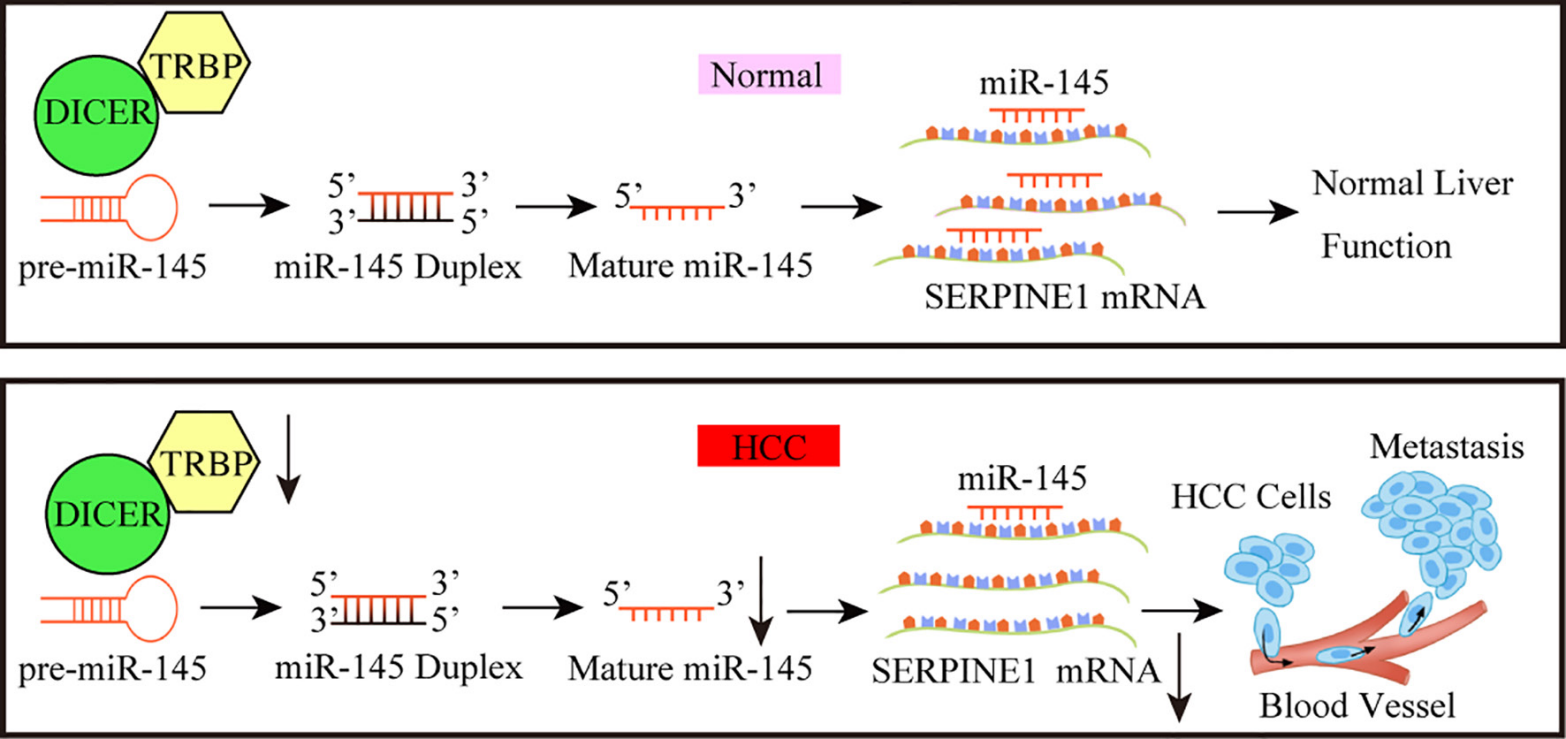
A working model for the TARBP2-miR-145-SERPINE1 axis in HCC progression. The loss of TARBP2 induces the upregulation of *SERPINE1* through reducing the processing of miR-145. This linear TARBP2-miR-145-SERPINE1 axis is involved in proliferation, migration, and invasion in HCC cells.

In conclusion, the role of TARBP*2* through mediation of miR-145 and a downstream player SERPINE1 was established in HCC progression, thus providing a new intervention pathway (e.g. HIF-1 pathway) that could be exploited to improve the clinical outcomes of HCC in the future (**Figure 8**). Further investigations to confirm the TRABP2-miR-145-SERPINE1 axis in independent HCC patients and clinical specimens, as well as animal models are warranted.

## Data Availability Statement

The datasets presented in this study can be found in online repositories. The names of the repository/repositories and accession number(s) can be found in the article/**Supplementary Material**.

## Ethics Statement

The studies involving human participants were reviewed and approved by Ethics Committee of Zhongnan Hospital, Wuhan University (Approval Number: 2017058). The patients/participants provided their written informed consent to participate in this study.

## Author Contributions

L-ML performed the experiments, collected data, and drafted the initial manuscript. CC, CZ, and ZZ were responsible for bioinformatic processing, and analysis of the RNA-Seq data. R-XR and J-TH provided materials and reagents and created cell models. H-LS prepared the published work by those from the original research group, specifically critical review, commentary or revision—including pre- or post-publication stages. WZ supervised bioinformatics and formal statistical analysis and helped data interpretation. S-ML was liable for oversight and leadership responsibility for the research activity planning and execution, including mentorship external to the core team. All authors contributed to the article and approved the submitted version.

## Funding

This research was supported by the National Research Foundation (81772276, 81902141) and provincial Natural Science Fund for Creative Research Groups (2019CFA018).

## Conflict of Interest

The authors declare that the research was conducted in the absence of any commercial or financial relationships that could be construed as a potential conflict of interest.

## Abbreviations

HCC, hepatocellular carcinoma; TRBP, transactivation response element RNA-binding protein; miRNA, microRNA; SERPINE1, serpin family E member 1; RNA-Seq, RNA-sequencing; BCLC, Barcelona clinic liver cancer; OS, overall survival; ROC, receiver operating characteristic; AUC, area under curve; KM, Kaplan-Meier; IHC, immunohistochemistry; PDK1, phosphoinositide-dependent protein kinase-1; EDN1, endothelin 1; ALT, alanine aminotransferase AST, aspartate aminotransferase; BUN, blood urea nitrogen; CREA, creatinine; TC, total cholesterol; TG, triglyceride; HDL-C, high-density lipoprotein cholesterol; LDL-C, low-density lipoprotein cholesterol; AFP, α-fetoprotein; CEA, carcinoembryonic antigen.
