# Corrigendum: Loss of TARBP2 Drives the Progression of Hepatocellular Carcinoma *via* miR-145-SERPINE1 Axis

**DOI:** 10.3389/fonc.2021.746958

**Published:** 2021-09-07

**Authors:** Li-Man Li, Chang Chen, Ruo-Xi Ran, Jing-Tao Huang, Hui-Lung Sun, Chang Zeng, Zhou Zhang, Wei Zhang, Song-Mei Liu

**Affiliations:** ^1^Department of Clinical Laboratory, Center for Gene Diagnosis, and Program of Clinical Laboratory Medicine, Zhongnan Hospital of Wuhan University, Wuhan, China; ^2^Department of Preventive Medicine, Northwestern University Feinberg School of Medicine, Chicago, IL, United States; ^3^Department of Clinical Laboratory, Renmin Hospital, Wuhan University, Wuhan, China; ^4^Department of Chemistry and Institute for Biophysical Dynamics, Howard Hughes Medical Institute, The University of Chicago, Chicago, IL, United States; ^5^Institute of Precision Medicine, Jining Medical University, Jining, China; ^6^Hubei Province Key Laboratory of Allergy and Immunology, Wuhan, China

**Keywords:** HCC, TARBP2, miR-145, SERPINE1, progression

In the original article, there were mistakes in **Figure 5** and **Figure 7** as published. In **Figure 5A** and **Figure 7A**, CTL should be sh-Ctrl to keep consistence of the abbreviation throughout of the manuscript. In **Figure 5B**, a layer was not removed due to our less carefulness during PS operation. The corrected Figures appear below.

**Figure 5:**
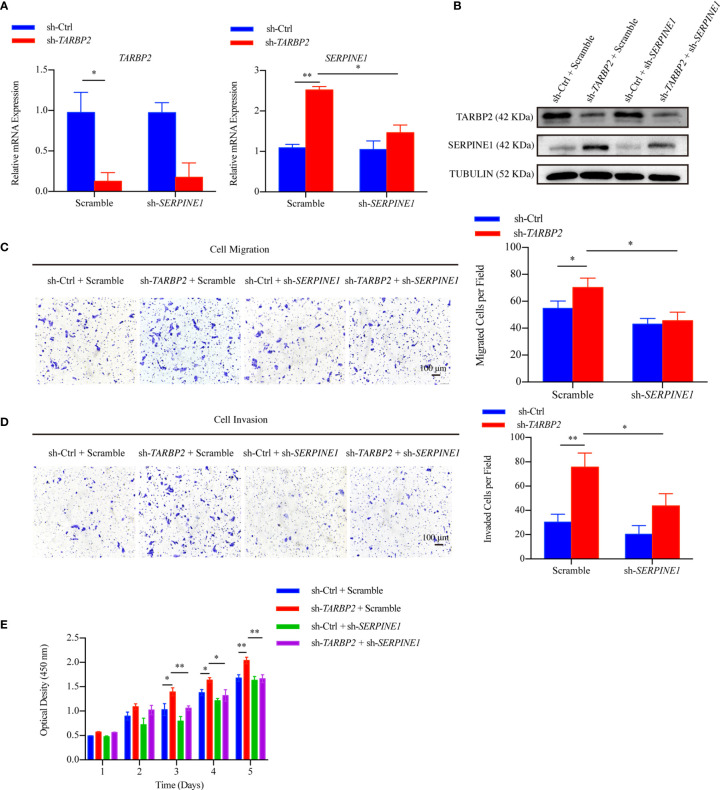
SERPINE1 is a downstream player of TARBP2. **(A, B)** The mRNA and protein expression of TARBP2 and SERPINE1 in the stable sh-TARBP2 HepG2 cells after treatment with scramble control and sh-SERPINE1. **(C)** Cell migration; **(D)** Invasion; and **(E)** Proliferation assays of sh-TARBP2 HepG2 cells treated with scramble control and sh-SERPINE1. *p-value < 0.05; **p-value < 0.01.

**Figure 7:**
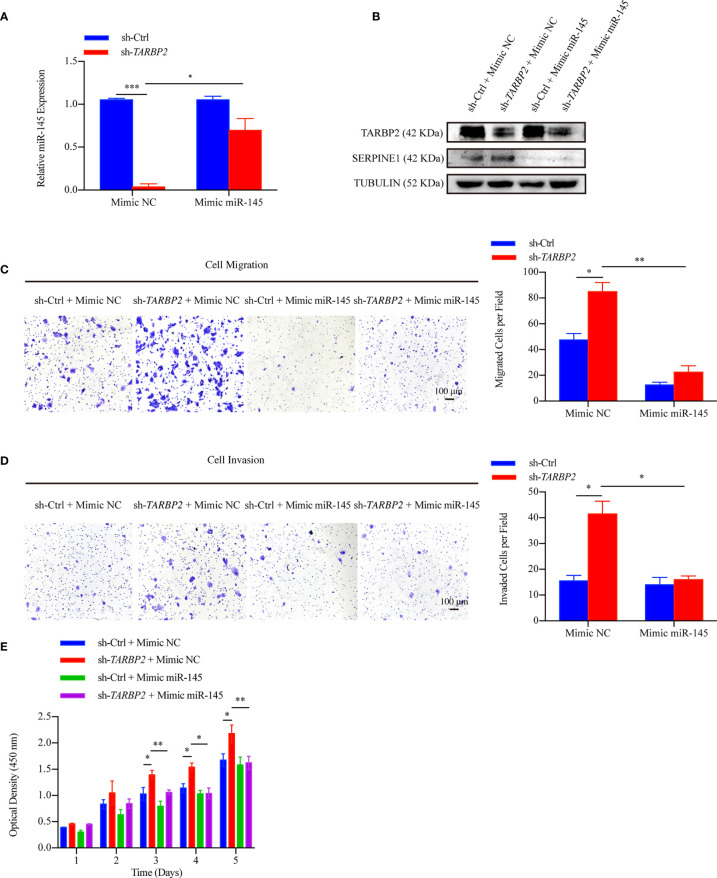
miR-145 mediates SERPINE1 to affect the role of TARBP2 in HCC progression. **(A)** Levels of miR-145 in sh-TARBP2 HepG2 cells after treatment with Mimic NC and Mimic miR-145. **(B)** Protein levels of SERPINE1 and TARBP2 in the stable sh-TARBP2 HepG2 cells after treatment with Mimic NC and Mimic miR-145. **(C)** Cell migration; **(D)** Invasion; and **(E)** Proliferation of sh-TARBP2 HepG2 cells treated with Mimic NC and Mimic miR-145. *p-value < 0.05; **p-value < 0.01; NC, negative control.

The authors apologize for this error and state that this does not change the scientific conclusions of the article in any way. The original article has been updated.

## Publisher’s Note

All claims expressed in this article are solely those of the authors and do not necessarily represent those of their affiliated organizations, or those of the publisher, the editors and the reviewers. Any product that may be evaluated in this article, or claim that may be made by its manufacturer, is not guaranteed or endorsed by the publisher.

