# Supplementary material for: Loss of TARBP2 Drives the Progression of Hepatocellular Carcinoma via miR-145-SERPINE1 Axis
Source: Front Oncol. 2021 Jun 24;11:620912. doi: 10.3389/fonc.2021.620912 (PMC8265608; doi:10.3389/fonc.2021.620912)
Supplement: Supplementary file 1 [file DataSheet_1.docx]

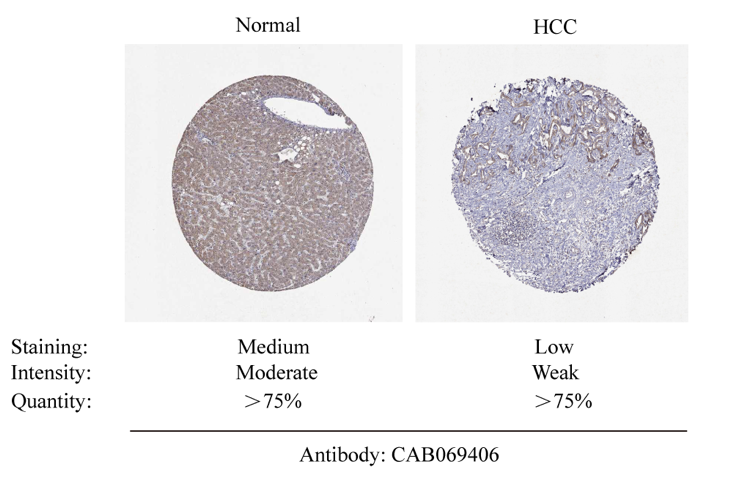


**Figure S1** Immunohistochemistry images of TARBP2 in normal liver and HCC tissues based on Human Protein Atlas database. The protein expression staining score is categorized into high, medium, low and not detected, reflecting the protein level of in this specimen.


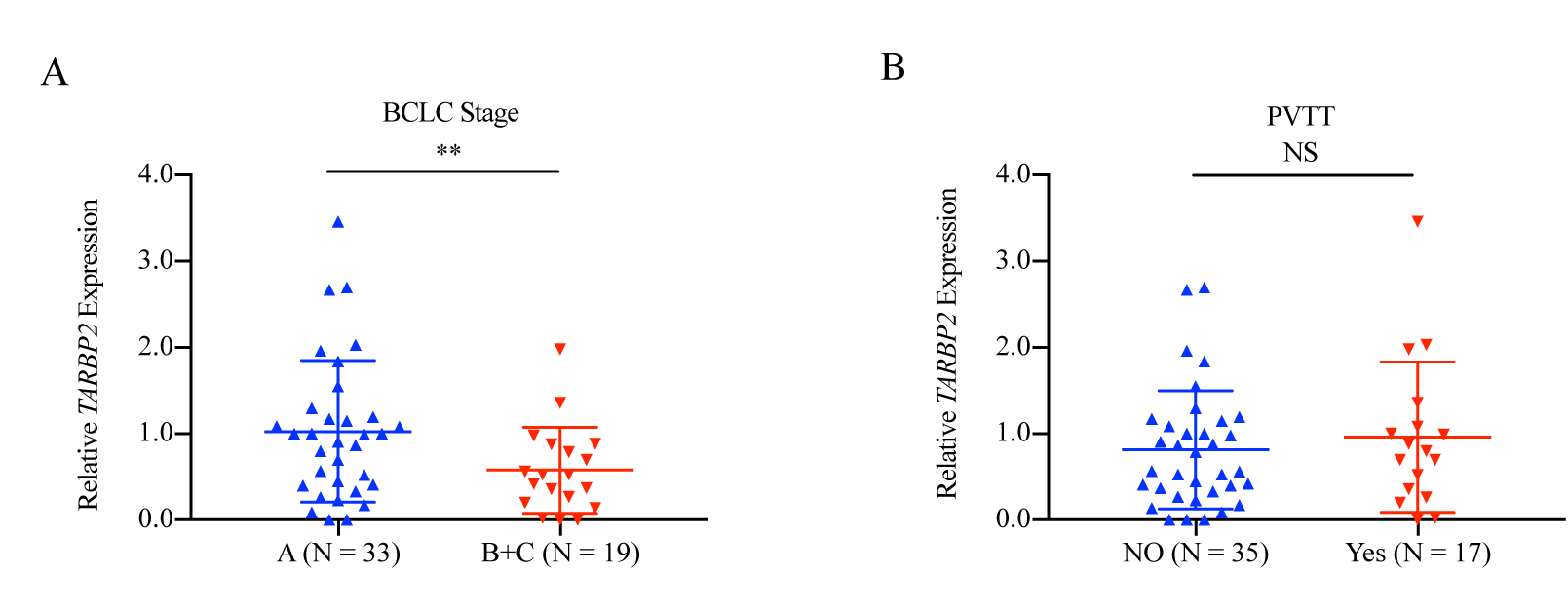


**Figure S2** TARBP2 expression in different BCLC stages of HCC patients (S1A) and in HCC patients with and without PVTT (S1B). BCLC, barcelona clinic liver cancer; PVTT, portal vein tumor thrombus.**p-value < 0.01; NS, no significance.


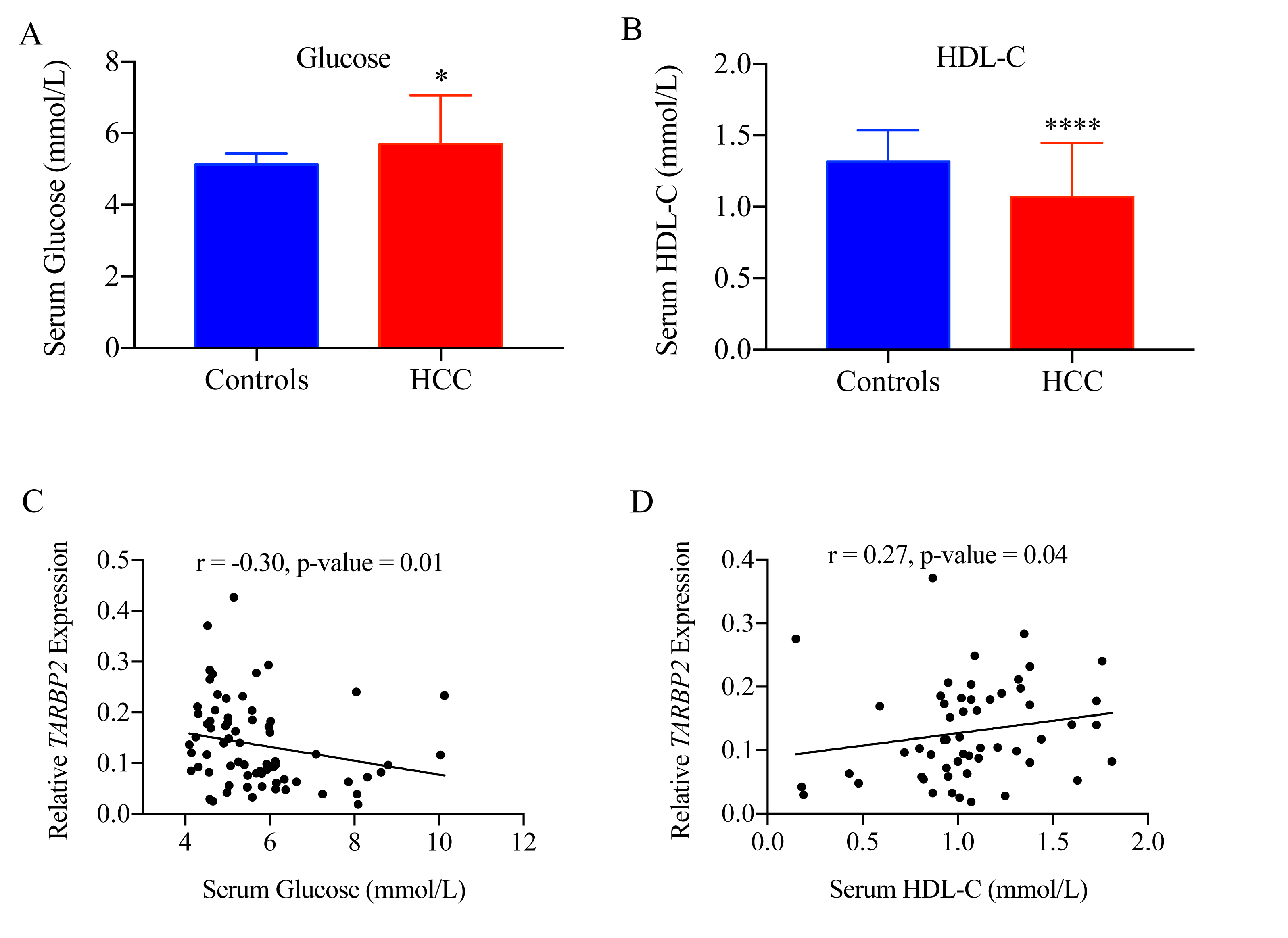


**Figure S3 Serum levels of glucose and HDL-C are associated with mRNA expression of *TARBP2* in white blood cells.** (S3A) Serum glucose in HCC was higher than healthy controls (Mann-Whitney test p-value = 0.049). (S1B) Serum HDL-C was lower relative to healthy controls (Mann-Whitney test p-value = 0.03×10-5). (S3C-D) The mRNA expression of *TARBP2* was negatively associated with glucose (Pearson r = -0.30, p-value = 0.01) and positively associated with HDL-C (Pearson r = 0.27, p-value = 0.04). *p-value < 0.05；****p-value < 0.0001.


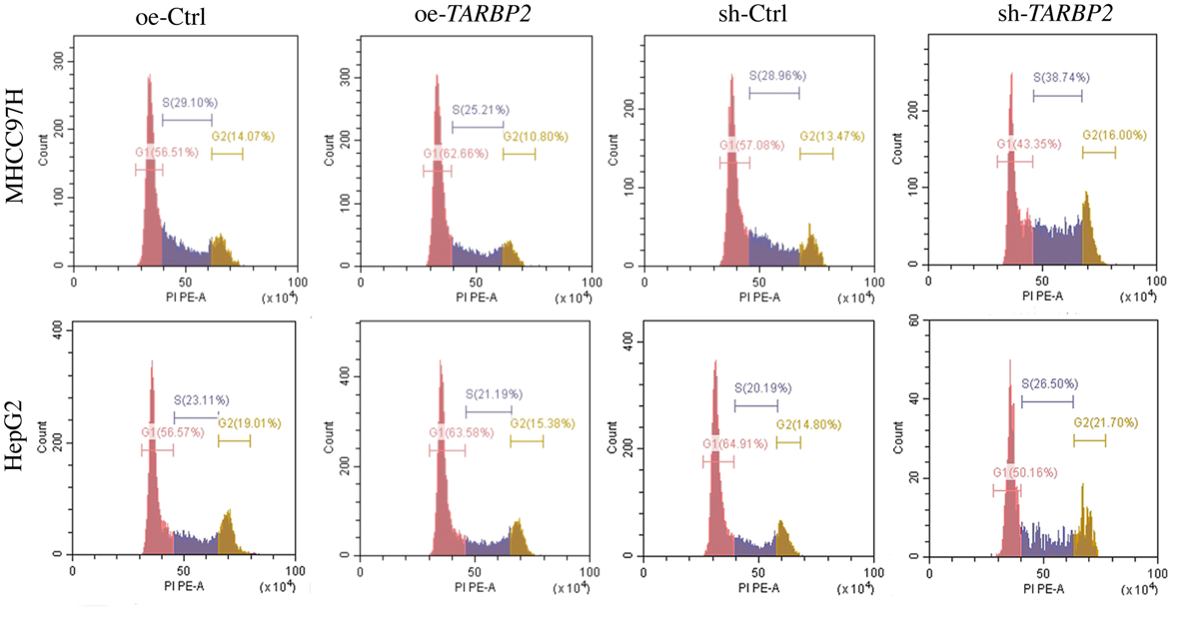

**Figure S4** Overexpression of *TARBP2* increases the proportion of HCC cells in G1 Phase, while *TARBP2* knockdown has a reverse effect. *p-value < 0.05; **p-value < 0.01; ***p-value < 0.001; NS, no significance.

**
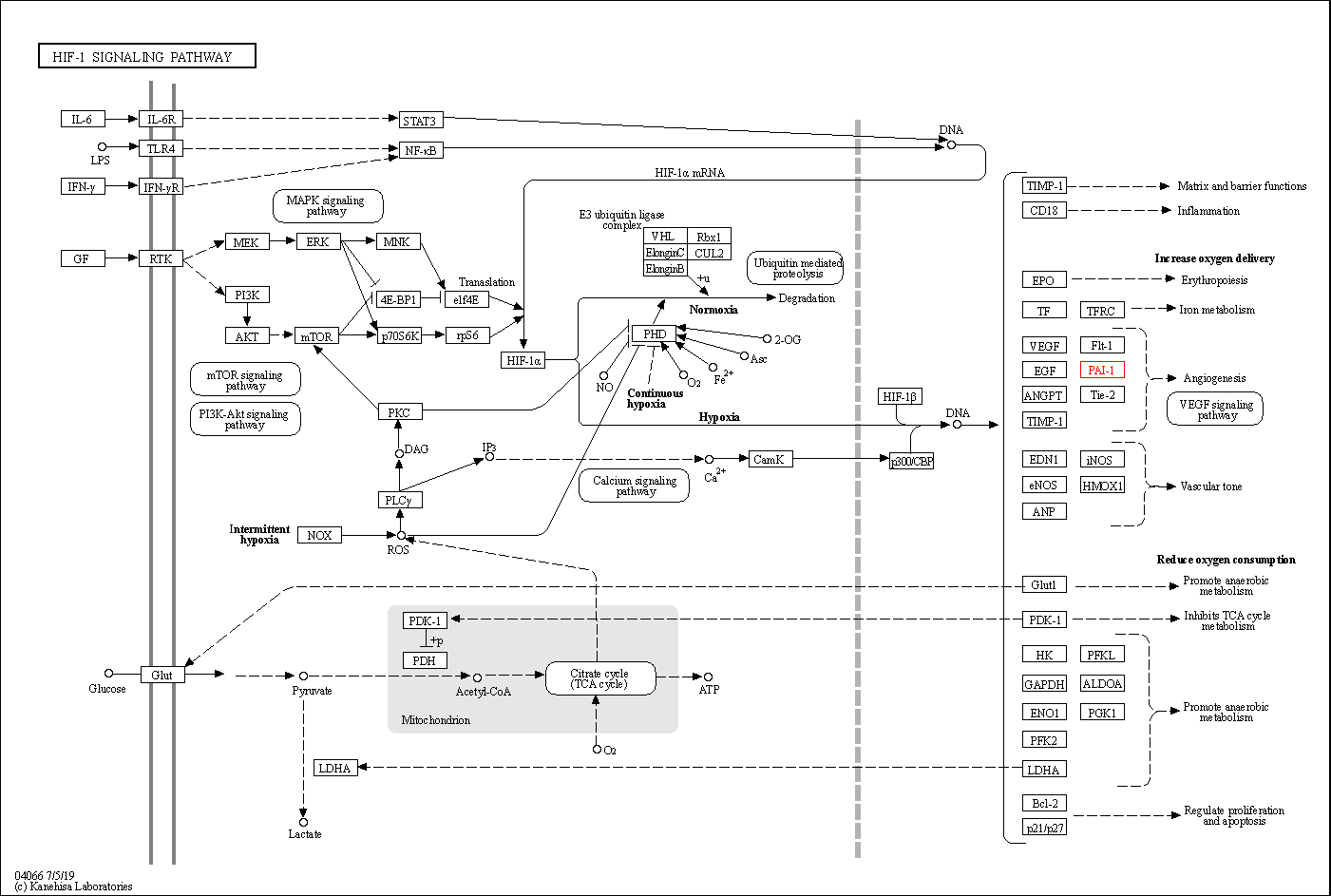
**

**Figure S5** SEPINE1 in HIF-1 pathway and SERPINE1 is in red.

**
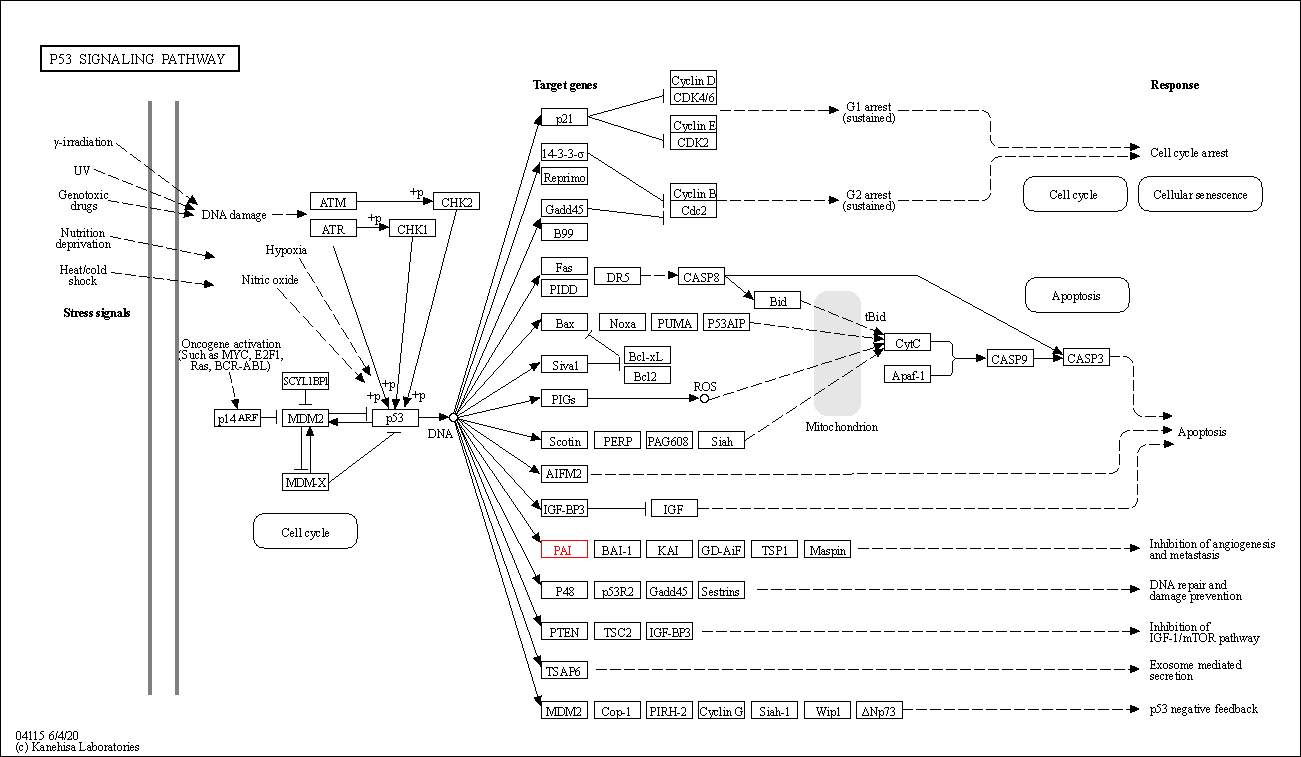
**

**Figure S6** SEPINE1 in P53 pathway and SERPINE1 is in red.

**Table S1.** Summary of cell lines used in this study

| Cell Lines | Characteristics | Reference |
| --- | --- | --- |
| L02 | Human normal hepatocyte cell line | (1, 2) |
| Hep3B | HCC cell line with hepatitis B virus background | (3) |
| SNU387 | HCC cell line with hepatitis B virus background, grade IV/V, pleomorphic hepatocellular carcinoma. | (4) |
| MHCC97H | HCC cell line with high metastatic potential | (5, 6) |
| HepG2 | Hepatocellular carcinoma/hepatoma Referencecell line/ hepatoblastoma cell line | (7) |
| HEK-293T | Embryonic kidney cell line for transfection | (8) |

**Table S2.** Primers for qRT-PCR

| Genes | Forward | Reverse |
| --- | --- | --- |
| *TARBP2* | 5’-AGGACATTCCGGTTTTTACTGC-3’ | 5’-CTGGGTCACTGTGTACTCCG-3’ |
| *PDK1* | 5’-GTGTAGATTAGAGGGATG-3’ | 5’-AAGGAATAGTGGGTTAGG-3’ |
| *SERPINE1* | 5’-GCAGCAGATTCAAGCAGCTATGG-3’ | 5’-GCGTCTGTGGTGCTGATCTCATC-3’ |
| *EDN1* | 5’-CTGGTTCCTGACTGGCAAAG-3’ | 5’-GGAAGCCAGTGAAGATGGTT-3’ |
| *GAPDH* | 5’-ATGACATCAAGAAGGTGGTG-3’ | 5’-ATGACATCAAGAAGGTGGTG-3’ |

*TARBP2*, transactivation response element RNA-binding protein; *PDK1*, pyruvate dehydrogenase kinase 1;

*SERPINE1*, serpin family E member 1; EDN1, endothelin 1.

**Table S3.** Correlation between mRNA expression of *TARBP2* and clinicopathologic parameters

| Parameter | Number of cases | *TARBP2* expression | |  |
| --- | --- | --- | --- | --- |
|  |  | Low (N = 26) | High (N = 26) | p-value |
| Gender |  |  |  |  |
| Male | 39 | 21 | 18 | 0.337 |
| Female | 13 | 5 | 8 |  |
| Age(years) |  |  |  |  |
| ＜65 | 40 | 18 | 22 | 0.188 |
| ≥65 | 12 | 8 | 4 |  |
| Smoking |  |  |  |  |
| Yes | 23 | 13 | 10 | 0.402 |
| No | 29 | 13 | 16 |  |
| Drinking |  |  |  |  |
| Yes | 20 | 12 | 8 | 0.254 |
| No | 32 | 14 | 18 |  |
| Tumor size |  |  |  |  |
| ＜5 cm | 18 | 8 | 10 | 0.706 |
| ≥5 cm | 34 | 16 | 16 |  |
| HBV infection |  |  |  |  |
| Positive | 39 | 22 | 17 | 0.109 |
| Negative | 13 | 4 | 9 |  |
| BCLC |  |  |  |  |
| A | 33 | 14 | 19 | 0.029* |
| B+C | 19 | 14 | 5 |  |
| Child-Pugh score |  |  |  |  |
| A | 42 | 20 | 22 | 0.482 |
| B | 10 | 6 | 4 |  |
| Liver cirrhosis |  |  |  |  |
| Yes | 17 | 9 | 8 | 0.768 |
| No | 35 | 17 | 18 |  |
| Serum AFP (mg/L) |  |  |  |  |
| ＜400 | 18 | 6 | 8 | 0.532 |
| ≥400 | 38 | 20 | 18 |  |
| PVTT |  |  |  |  |
| Yes | 17 | 13 | 4 | 0.036* |
| No | 35 | 16 | 19 |  |

BCLC, barcelona clinic liver cancer; PVTT, portal vein tumor thrombus.

**Table S4.** Clinical characteristics of blood sample derived population

| Parameters | Controls (N = 70) | HCC (N = 86) | p-value |
| --- | --- | --- | --- |
| Gender, (male/female) | 56/14 | 60/26 | 0.145 |
| Age, (years)^$^ | 55.13 (10.48) | 56.6 (10.32) | 0.608 |
| ALT, U/L^&^ | 17 (14.00-21.00) | 40 (24.00-61.75) | 1.51×10^-12^ |
| AST, U/L^&^ | 20 (18.00-24.00) | 50 (28.00-89.75) | 4.27×10^-17^ |
| Glucose, mmol/L^&^ | 5.14 (4.96-5.30) | 5.44 (4.65-6.12) | 0.049 |
| Blood Urea Nitrogen, mmol/L^$^ | 5.07 (1.08) | 5.25 (1.89) | 0.636 |
| Creatinine, μmol/L^&^ | 69.1 (61.5-81.9) | 72 (61.8-83.9) | 0.808 |
| Uric acid, μmol/L^$^ | 311.79 (67.92) | 306.14 (96.32) | 0.635 |
| Total cholesterol, mmol/L^$^ | 4.48 (0.50) | 3.73 (1.16) | 0.004 |
| Triglycerides, mmol/L^&^ | 0.99 (0.81-1.32) | 1.04 (0.68-1.28) | 0.428 |
| HDL cholesterol, mmol/L^&^ | 1.28 (1.15-1.43) | 1.04 (0.92-1.32) | 3.00×10^-7^ |
| LDL cholesterol, mmol/^$^ | 2.5 (0.50) | 2.37 (0.90) | 0.819 |
| AFP, ng/mL^&^ | 2.829 (2.22-4.17) | 20.29 (3.19-1508.8) | 7.00×10^-6^ |
| CEA, ng/mL^&^ | 1.60 (1.09-2.70) | 2.37(1.84-3.01) | 0.001 |

^$^ Normally distributed data are described as mean (std. deviation); ^&^Non-normally distributed

data are expressed as median (interquartile range); ALT, alanine aminotransferase;

AST, aspartate aminotransferase; HDL, high-density Lipoprotein; LDL, low-density lipoprotein;

AFP, α-fetoprotein; CEA, carcinoembryonic antigen.

**Table S5.** KEGG pathways for Intersect Genes of oe-Ctrl vs oe-*TARBP2* and sh-Ctrl vs sh-*TARBP2* based on different levels of cut-offs

| Term | Count | p-value | FDR | Genes |
| --- | --- | --- | --- | --- |
| **Differential genes at 50% cut-off** |  |  |  |  |
| Insulin secretion | 8 | 0.015 | 18.150 | *GPR119, KCNN1, GCK, ADCY5, ATP1A3, PDX1, CACNA1C, CAMK2A* |
| Type II diabetes mellitus | 6 | 0.015 | 18.163 | *TNF, GCK, PIK3CA, PDX1, CACNA1C, PIK3R2* |
| Cholinergic synapse | 9 | 0.021 | 23.740 | *ACHE, GNAO1, FYN, ADCY5, PIK3CA, CACNA1C, CAMK2A, ITPR2, PIK3R2* |
| Wnt signaling pathway | 10 | 0.026 | 29.032 | *WNT5A, WNT10A, CTBP2, SFRP1, VANGL2, WNT9B, MMP7, SOX17, CAMK2A, DAAM2* |
| HIF-1 signaling pathway | 8 | 0.028 | 30.754 | *PDK1, EDN1, SERPINE1, EGLN3, PIK3CA, TLR4, CAMK2A, PIK3R2* |
| mTOR signaling pathway | 6 | 0.032 | 34.512 | *TNF, RPS6KA2, PIK3CA, RICTOR, RRAGD, PIK3R2* |
| Pathways in cancer | 20 | 0.033 | 35.435 | *WNT5A, CEBPA, WNT10A, COL4A1, CTBP2, ADCY5, ARNT2, EGLN3, FGF11, BIRC7, FGF22, FGF21, MMP1, CXCR4, LPAR6, WNT9B, PIK3CA, GSTP1, ITGA2B, PIK3R2* |
| Chagas disease (American trypanosomiasis) | 8 | 0.041 | 41.609 | *GNAO1, TNF, CD247, SERPINE1, PIK3CA, TLR4, PPP2R2C, PIK3R2* |
| cAMP signaling pathway | 12 | 0.042 | 42.394 | *GPR119, TIAM1, ADCY5, GRIN1, ATP1A3, PIK3CA, ARAP3, HTR1D, CACNA1C, CAMK2A, HCAR2, PIK3R2* |
| Dopaminergic synapse | 9 | 0.044 | 43.606 | *GNAO1, CALY, ADCY5, KIF5C, TH, CACNA1C, CAMK2A, PPP2R2C, ITPR2* |
| Proteoglycans in cancer | 12 | 0.045 | 44.279 | *WNT5A, WNT10A, CAV2, TNF, CD44, TIAM1, WNT9B, PIK3CA, TLR4, CAMK2A, ITPR2, PIK3R2* |
| Mineral absorption | 5 | 0.046 | 45.077 | *MT1M, MT1A, CYBRD1, ATP1A3, MT1X* |
| Platelet activation | 9 | 0.047 | 46.167 | *FYN, ADCY5, MYLK2, PIK3CA, GP1BA, COL5A2, ITPR2, ITGA2B, PIK3R2* |
| ECM-receptor interaction | 7 | 0.051 | 49.110 | *ITGA9, COL4A1, CD44, RELN, GP1BA, COL5A2, ITGA2B* |
| Amphetamine addiction | 6 | 0.052 | 49.665 | *ARC, ADCY5, GRIN1, TH, CACNA1C, CAMK2A* |
|  |  |  |  |  |
| **Differential genes at 60% cut-off** |  |  |  |  |
| cAMP signaling pathway | 11 | 0.008 | 9.599 | *GPR119, TIAM1, ADCY5, GRIN1, ATP1A3, PIK3CA, HTR1D, CACNA1C, CAMK2A, HCAR2, PIK3R2* |
| Cholinergic synapse | 8 | 0.008 | 9.737 | *ACHE, GNAO1, FYN, ADCY5, PIK3CA, CACNA1C, CAMK2A, PIK3R2* |
| Insulin secretion | 7 | 0.008 | 9.879 | *GPR119, GCK, ADCY5, ATP1A3, PDX1, CACNA1C, CAMK2A* |
| Amphetamine addiction | 6 | 0.012 | 13.718 | *ARC, ADCY5, GRIN1, TH, CACNA1C, CAMK2A* |
| HIF-1 signaling pathway | 7 | 0.015 | 16.796 | *PDK1, EDN1, SERPINE1, EGLN3, PIK3CA, CAMK2A, PIK3R2* |
| Dopaminergic synapse | 8 | 0.017 | 19.294 | *GNAO1, CALY, ADCY5, KIF5C, TH, CACNA1C, CAMK2A, PPP2R2C* |
| Type II diabetes mellitus | 5 | 0.017 | 19.501 | *GCK, PIK3CA, PDX1, CACNA1C, PIK3R2* |
| Melanogenesis | 7 | 0.018 | 19.864 | *WNT5A, WNT10A, GNAO1, ADCY5, EDN1, WNT9B, CAMK2A* |
| Platelet activation | 8 | 0.018 | 20.682 | *FYN, ADCY5, MYLK2, PIK3CA, GP1BA, COL5A2, ITGA2B, PIK3R2* |
| Wnt signaling pathway | 8 | 0.025 | 26.747 | *WNT5A, WNT10A, SFRP1, VANGL2, WNT9B, MMP7, CAMK2A, DAAM2* |
| Adrenergic signaling in cardiomyocytes | 8 | 0.025 | 26.747 | *RPS6KA5, ADCY5, CACNG6, ATP1A3, SCN4B, CACNA1C, CAMK2A, PPP2R2C* |
| Focal adhesion | 10 | 0.027 | 29.392 | *ITGA9, CAV2, COL4A1, RASGRF1, FYN, MYLK2, PIK3CA, COL5A2, ITGA2B, PIK3R2* |
| Pathways in cancer | 15 | 0.033 | 34.623 | *WNT5A, CEBPA, WNT10A, COL4A1, CXCR4, ADCY5, ARNT2, WNT9B, FGF11, EGLN3, PIK3CA, MMP1, GSTP1, PIK3R2, ITGA2B* |
| ECM-receptor interaction | 6 | 0.034 | 35.457 | *ITGA9, COL4A1, CD44, GP1BA, COL5A2, ITGA2B* |
| Aldosterone-regulated sodium reabsorption | 4 | 0.047 | 45.026 | *NR3C2, ATP1A3, PIK3CA, PIK3R2* |
| Circadian entrainment | 6 | 0.047 | 45.507 | *RPS6KA5, GNAO1, ADCY5, GRIN1, CACNA1C, CAMK2A* |
|  |  |  |  |  |
| **Differential genes at 70% cut-off** |  |  |  |  |
| Wnt signaling pathway | 8 | 0.004 | 5.146 | *WNT5A, WNT10A, SFRP1, VANGL2, WNT9B, MMP7, CAMK2A, DAAM2* |
| Dopaminergic synapse | 7 | 0.012 | 13.468 | *GNAO1, CALY, KIF5C, TH, CACNA1C, CAMK2A, PPP2R2C* |
| HIF-1 signaling pathway | 6 | 0.014 | 15.718 | *PDK1, EDN1, SERPINE1, EGLN3, CAMK2A, PIK3R2* |
| Melanogenesis | 6 | 0.016 | 18.239 | *WNT5A, WNT10A, GNAO1, EDN1, WNT9B, CAMK2A* |
| Adrenergic signaling in cardiomyocytes | 7 | 0.016 | 18.470 | *RPS6KA5, CACNG6, ATP1A3, SCN4B, CACNA1C, CAMK2A, PPP2R2C* |
| Insulin secretion | 5 | 0.037 | 37.336 | *GPR119, GCK, ATP1A3, CACNA1C, CAMK2A* |
| Hepatitis C | 6 | 0.047 | 45.028 | *IFIT1, TLR3, PPP2R2C, CLDN23, CLDN14, PIK3R2* |
|  |  |  |  |  |
| **Differential genes at 80% cut-off** |  |  |  |  |
| HIF-1 signaling pathway | 6 | 0.002 | 1.830 | *PDK1, EDN1, SERPINE1, EGLN3, CAMK2A, PIK3R2* |
| Dopaminergic synapse | 6 | 0.005 | 6.250 | *GNAO1, CALY, KIF5C, TH, CACNA1C, CAMK2A* |
| Cholinergic synapse | 5 | 0.017 | 18.243 | *GNAO1, FYN, CACNA1C, CAMK2A, PIK3R2* |

**References**：

1. Huang J, Wang Y, Guo Y, Sun S. Down-regulated microRNA-152 induces aberrant DNA methylation in hepatitis B virus-related hepatocellular carcinoma by targeting DNA methyltransferase 1. Hepatology 2010;52:60-70.

2. Huang F, Zheng X, Ma X, Jiang R, Zhou W, Zhou S, Zhang Y, et al. Theabrownin from Pu-erh tea attenuates hypercholesterolemia via modulation of gut microbiota and bile acid metabolism. Nat Commun 2019;10:4971.

3. Knowles BB, Howe CC, Aden DP. Human hepatocellular carcinoma cell lines secrete the major plasma proteins and hepatitis B surface antigen. Science 1980;209:497-499.

4. Park JG, Lee JH, Kang MS, Park KJ, Jeon YM, Lee HJ, Kwon HS, et al. Characterization of cell lines established from human hepatocellular carcinoma. Int J Cancer 1995;62:276-282.

5. Li Y, Tang Y, Ye L, Liu B, Liu K, Chen J, Xue Q. Establishment of a hepatocellular carcinoma cell line with unique metastatic characteristics through in vivo selection and screening for metastasis-related genes through cDNA microarray. J Cancer Res Clin Oncol 2003;129:43-51.

6. Wang FB, Rong Y, Fang M, Yuan JP, Peng CW, Liu SP, Li Y. Recognition and capture of metastatic hepatocellular carcinoma cells using aptamer-conjugated quantum dots and magnetic particles. Biomaterials 2013;34:3816-3827.

7. Aden DP, Fogel A, Plotkin S, Damjanov I, Knowles BB. Controlled synthesis of HBsAg in a differentiated human liver carcinoma-derived cell line. Nature 1979;282:615-616.

8. Pear WS, Nolan GP, Scott ML, Baltimore D. Production of high-titer helper-free retroviruses by transient transfection. Proc Natl Acad Sci U S A 1993;90:8392-8396.
